# Identification and Rational Redesign of Peptide Ligands to CRIP1, A Novel Biomarker for Cancers

**DOI:** 10.1371/journal.pcbi.1000138

**Published:** 2008-08-01

**Authors:** Jihua Hao, Adrian W. R. Serohijos, Gail Newton, Gina Tassone, Zuncai Wang, Dennis C. Sgroi, Nikolay V. Dokholyan, James P. Basilion

**Affiliations:** 1 Department of Radiology, Case Western Reserve University, Cleveland, Ohio, United States of America; 2 Department of Biochemistry and Biophysics, University of North Carolina at Chapel Hill, Chapel Hill, North Carolina, United States of America; 3 Department of Physics and Astronomy, University of North Carolina at Chapel Hill, Chapel Hill, North Carolina, United States of America; 4 Harvard Medical School, Massachusetts General Hospital, Boston, Massachusetts, United States of America; 5 Lineberger Comprehensive Cancer Center, University of North Carolina at Chapel Hill, Chapel Hill, North Carolina, United States of America; 6 Department of Biomedical Engineering, Case Western Reserve University, Cleveland, Ohio, United States of America; 7 National Foundation for Cancer Research Center for Molecular Imaging, Case Western Reserve University, Cleveland, Ohio, United States of America; Harvard University, United States of America

## Abstract

Cysteine-rich intestinal protein 1 (CRIP1) has been identified as a novel marker for early detection of cancers. Here we report on the use of phage display in combination with molecular modeling to identify a high-affinity ligand for CRIP1. Panning experiments using a circularized C7C phage library yielded several consensus sequences with modest binding affinities to purified CRIP1. Two sequence motifs, A1 and B5, having the highest affinities for CRIP1, were chosen for further study. With peptide structure information and the NMR structure of CRIP1, the higher-affinity A1 peptide was computationally redesigned, yielding a novel peptide, A1M, whose affinity was predicted to be much improved. Synthesis of the peptide and saturation and competitive binding studies demonstrated approximately a 10–28-fold improvement in the affinity of A1M compared to that of either A1 or B5 peptide. These techniques have broad application to the design of novel ligand peptides.

## Author Summary

Breast cancer is one of the most frequently diagnosed malignancies in American females and is the second leading cause of cancer deaths in women. Several improvements in diagnostic protocols have enhanced our ability for earlier detection of breast cancer, resulting in improvement of therapeutic outcome and an increased survival rate for breast cancer patients. However, current early screening techniques are neither comprehensive nor infallible. Imaging techniques that improve breast cancer detection, localization, and evaluation of therapy are essential in combating the disease. Cysteine-rich intestinal protein 1 (CRIP1) has been identified as a novel marker for early detection of breast cancers. Here, we report the use of phage display and computational molecular modeling to identify a high-affinity ligand for CRIP1. Phage display panning experiments initially identified consensus peptide sequences with modest binding affinity to purified CRIP1. Using ab initio modeling of binding peptide structures, computational docking, and recently developed free energy estimation protocols, we redesigned the peptides to increase their affinity for CRIP1. Synthesis of the redesigned peptide and binding studies demonstrated approximately a 10–28-fold improvement in the binding affinity. The combination of computational and experimental techniques in this study demonstrates a potentially powerful tool in modulating protein–protein interactions.

## Introduction

Cysteine-rich intestinal protein 1 (CRIP1) belongs to the LIM/double zinc finger protein family, which includes cysteine- and glycine-rich protein-1, rhombotin-1, rhombotin-2, and rhombotin-3. Human CRIP1, primarily a cytosolic protein, was cloned in 1997 [1] using RT-PCR of human small intestine RNA and oligonucleotides whose sequence was derived from the human heart homolog of this protein, CRHP [2]. Recently CRIP1 has been identified as a very exciting biomarker for human breast cancers [3],^4^, cervical cancers [5],[6], pancreatic cancers [7],[8] and potentially other cancers [4],[9]. In experiments comparing CRIP1 expression in human breast cancer to matched normal breast tissue the mRNA for this target was overexpressed 8–10-fold in approximately 90% of both invasive and ductal carcinoma in situ [3]. Furthermore, in situ hybridization studies demonstrated close association of the expression with the ductal carcinoma cells [3]. CRIP1 overexpression has also been demonstrated to be the most highly differentially expressed gene in invasive cervical carcinomas; 100-fold up-regulation relative to normal cervical keratinocytes measured in 34 cervical tissues from different clinically defined stages [5],[6]. CRIP1 was also found to have high levels of expression in pancreatic adenocarcinoma, lung cancers and colorectal cancers [7]–[9]. These data strongly support the development of imaging probes targeting CRIP1 to improve cancer detection.

Phage display technology is a robust methodology for identifying peptides that bind relatively tightly to target proteins. This is especially true if the targeted protein's function is to bind peptides in vivo. In these applications, the first generation peptides have a generally lower *K*_d_ (10–100 µM) for their target and typically need to be structurally altered to improve binding before the peptides exhibit robust binding suitable to image the target protein. If structural data for the targeted protein exists, it should be feasible to utilize the data to help redesign in silico the Phage display-identified peptides thereby increasing their binding affinity. This approach is much more cost efficient than exhaustive screening of structured phage libraries or expansion of screening assays to include other types of phage display libraries.

Despite the potential utility of CRIP1 [10] as an imaging target, significant efforts to develop CRIP1-specific ligands have not been attempted. Here we utilized phage display techniques [11]–[20] to identify peptide ligands with micromolar binding affinity for purified human CRIP1 and exploited rational protein redesign [21]–[27] to increase the peptide's binding affinity. This approach has yielded a peptide that has approximately 10–28-fold improved binding affinity as measured by *in vitro* saturation and competitive binding assays. This study is a significant advance to the ultimate goal of synthesizing imaging probes that report CRIP1 expression levels in vivo.

## Results

We used phage display technology to identify peptides that bind relatively tightly to CRIP1. Then, we utilized NMR structural data of CRIP1 and computational methods to increase the peptide binding affinity to CRIP1.

### Expression of CRIP1 and Identification of Binding Peptides

CRIP1 was initially cloned into a mammalian expression vector and subsequently into pHAT10 for expression in bacteria. The pHAT10/CRIP1 vector encodes a naturally occurring polyhistidine epitope tag with the sequence of nonadjacent histidines that enable purification of expressed proteins under native conditions at neutral pH 7.0 (details of construct can be found in Figure S1 and Figure S2). Bacterial expression was chosen since it is a robust expression system and presumably CRIP1 does not require post-translational modifications for function. Cultures derived from these bacteria were induced to express CRIP1 using IPTG. We then isolated purified CRIP1 (see Methods). SDS-PAGE analysis of the cell lysate and fractions containing eluted CRIP1 show a single band for chimeric CRIP1 running approximately at the calculated molecular weight for the chimeric protein, 12.8 KDa (Figure S2 and Table S1). The yield of CRIP1 protein was approximately 10 mg of recombinant protein per liter of culture.

In order to generate CRIP1 protein that was as similar as possible to endogenous CRIP1, enterokinase cleavage was performed on purified CRIP1. Uncleaved contaminating HIS-tagged CRIP1 as well as HIS-tagged peptides were removed by re-running the digest over the CellThru resin and retaining the flow thru. This manipulation of the chimeric protein resulted in a polypeptide almost completely devoid of other “non-CRIP1” amino acids and was used as the bait for phage display studies. After four rounds of positive selection against enterokinase-truncated CRIP1, 29 phage DNA inserts were sequenced using a 96 gIII primer (5′-^HO^CCC TCA TAG TTA GCG TAA CG-3′). Sequencing verified that 18 of the 29 phagotopes were from the cysteine-constrained phage library, Table 1. Many of the peptide sequences contained similar motifs and six sequences occurred in more than one phagotope. The peptides A1 and C5 were identified four times, C1 three times, and A9, B1, and B5 twice. However, even accounting for conserved amino acid substitutions, no clear motif could be identified.

**Table 1:** Peptide sequences that were enriched after phage display panning experiments.

ID	Sequence	Frequency
A1^a^	LKDNHRS	4
A3	SVPINDS	1
A5	DHRQGSS	1
A6	APYNTLA	1
A8	SPHIIAS	1
A9^a^	MLHAYAQ	2
B1^a^	FLGFSQQ	2
B2	YDPIWRT	1
B3	FSTNMKT	1
B4	RTTGAQT	1
B5^a^	YDPIWRT	2
B7	PLFKGMS	1
B9	LPAYSTY	1
B10	RDSSAHQ	1
C1^a^	CYTAALA	3
C2	HANFLHM	1
C5^a^	TPRQSPI	4
C9	SLNTRSQ	1

a The sets of identical sequences derived from different phage clones.

To select clones for further analysis, we used ELISA to determine the relative binding affinities of selected individual phage clones to purified CRIP1 (see Methods and Figure S3). For these studies the purified chimeric CRIP1 was not reacted with enterokinase. Clone A1 and Clone B5 were measured to possess higher relative affinity. Although it occurred with the same frequency as clone A1, clone C5 exhibited lower binding affinity. With these results, inserts from the two clones with the highest affinities (A1 and B5) were further investigated as potential ligands to CRIP1.

### Molecular Modeling

The computational optimization of the binding affinity of the peptide initially identified from phage display involved three stages. We first constructed a structural model of the cyclic peptide A1, and then we identified putative binding sites on CRIP1 by docking. Lastly, we searched for new peptide sequences that optimize the stability of the peptide-CRIP1 complex.

**Figure 1:**
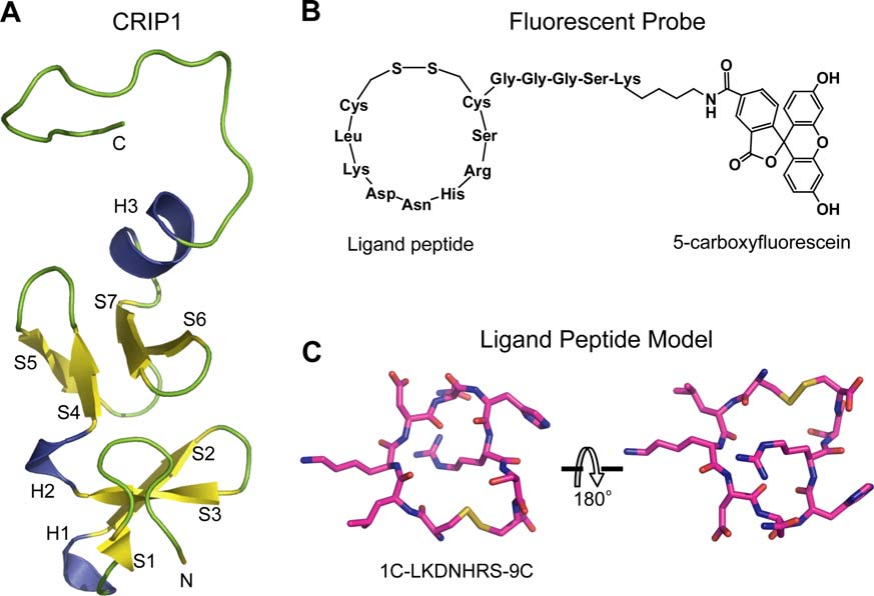
CRIP1 and the designed biomarker. (A) CRIP1 is composed of 2 LIM domains and a C-terminal loop that is unstructured. (B) The designed CRIP1 probe consists of a cyclic ligand peptide with a fluorescent molecule. (C) Cyclic peptide model corresponding to A1 derived from phage display experiments.

**Figure 2:**
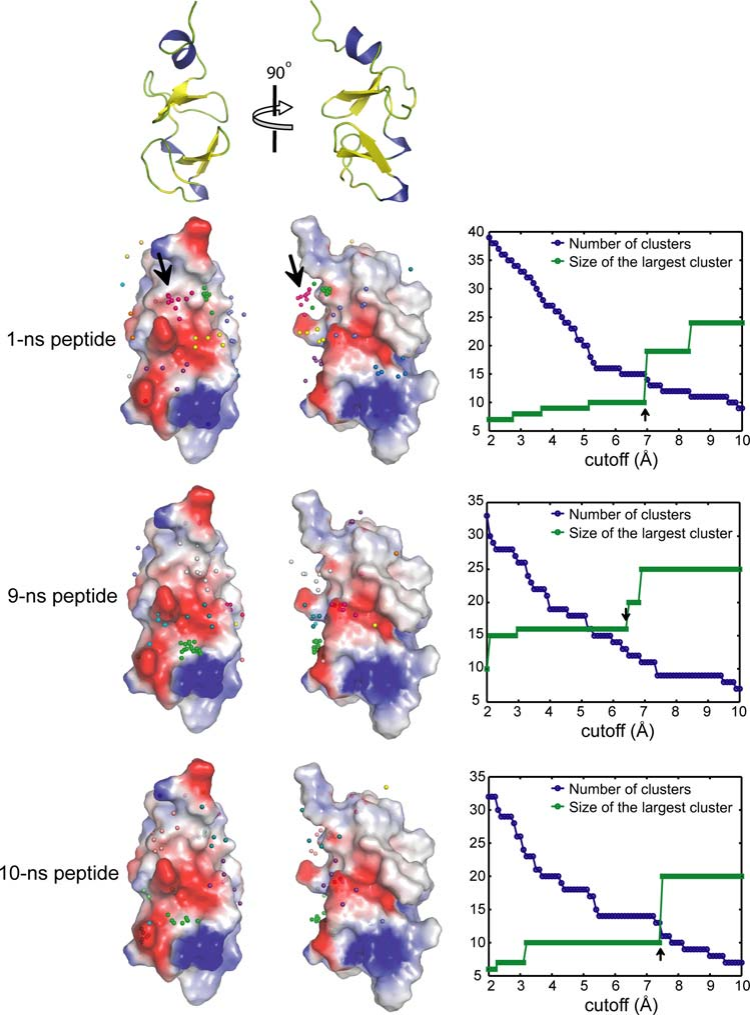
Putative binding sites on CRIP1. Three peptide models 1-ns, 2-ns, and 3-ns were docked onto the CRIP1 structure. The centers of mass of each peptide's C_α_ atoms are shown as spheres on the CRIP1 surface. The binding poses of each peptide model were clustered to determine putative binding sites. Spheres that belong to the same cluster are colored similarly. The largest cluster of docked 1-ns peptides, which is also the binding site of A1M (Figure 3), is shown with an arrow. On the left panel, we plot the number of clusters and the size of the largest cluster to determine the optimal cutoff for clustering. The final cutoff used in the clustering is shown by an arrow.

**Figure 3:**
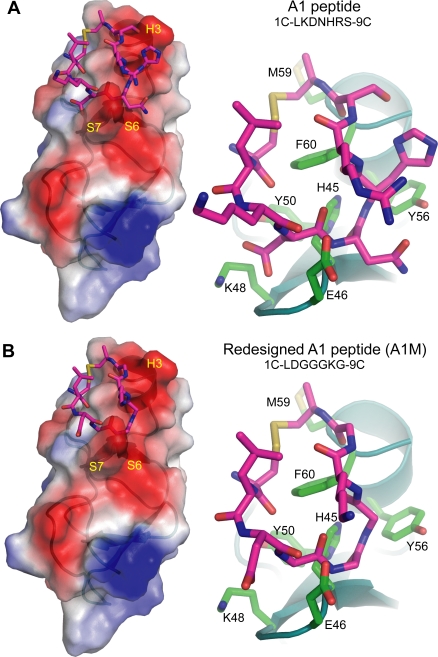
Peptide Redesign. (A) A1 peptide docked to groove formed by the S6–S7 turn and the helix H3 (left panel). CRIP 1 residues that form the putative A1 binding site (right panel). (B) Redesigned A1 (A1M) peptide that is predicted to have a higher affinity to CRIP1.

Shown in Figure 1C is the molecular model of the cyclic peptide A1 (see Methods). To remove the bias on the docking that may be introduced by using only one backbone peptide conformation, we first generated several peptide backbone conformations from snapshots of equilibrium molecular dynamics simulations. Each peptide was docked to the 48 conformations of CRIP1 derived from NMR [10]. By clustering the location of the peptides on the CRIP1 surface, we were able to identify and rank the putative binding sites (Figure 2). Interestingly, the peptides preferably bind to one face of CRIP1 (Figure 2). This side of CRIP1 contains two grooves, one formed by helix H3 and S6–S7 loop and another by S2–S3 loop and the N-terminal loop. The binding site of the successfully redesigned A1M is formed by Glu46, His45, Phe60, Tyr56, and Lys48 (Figure 3).

In the second stage of the redesign, we searched for peptide sequences that optimized the binding free energies of the peptide-CRIP1 complexes using heuristic algorithms and a physical force-field (see Methods). The methodology employs rapid side-chain packing and backbone relaxation to calculate the free energy change due to a mutation. For a given CRIP1-peptide complex, we determined a set of mutations in the bound peptide that resulted in the lowest free energy change, and thus, the highest predicted increase in binding affinity. All CRIP1-peptide complexes were subjected to redesign. All redesigned peptides were then grouped according to their starting peptide backbone conformation (1-ns, 9-ns, or 10-ns), and according to their putative binding site. The redesigned sequence CLDGGGKGC, which we denote here as A1M (“modified A1”), corresponds to a peptide with the lowest binding free energy ΔΔG among the redesigned sequences in the highest-ranked binding mode. In Table 2 we list representative peptide sequences with high binding affinity but located in other putative binding sites and featuring backbone conformations other than the 1-ns.

**Table 2:** Peptide sequences determined by molecular modeling.

Design ID	Sequence	ΔΔ*G* (kcal/mol)	Peptide model	Binding mode rank^a^
M1	CLDGGGKGC	−83	1-ns	1
M2	CLGGEKGGC	−63	10-ns	4.5^b^
M3	CGNDAGLGC	−55	10-ns	4.5
M4	CVGNSEPGC	−26	9-ns	8
M5	CGDKKQGGC	−24	9-ns	4.5

All docked peptide structures were subjected to the redesign protocol. Shown below are representative redesigned sequences from different peptide backbone conformations (1-ns, 9-ns, or 10-ns) and from different binding sites. The peptide A1M (CLDGGGKGC) exhibited both a high binding mode rank and a low ΔΔ*G* value; thus we select it for experimental testing.

a Binding mode rank pertains to the order of the cluster size to which the peptide complex belongs. A binding mode of rank 1 implies that the peptide is positioned on the site to which most other structures are also docked.

b M2 and M3 come from different binding sites but with equal number of docked peptides.

To identify the dominant motifs in the redesigned peptides, we show in Figure S5 the dominant sequence motifs in the top three candidates binding sites for each peptide model. There is a prevalence of Gly, presumably due to the strongly curved backbone that prefers more flexible Gly over any other residue when the peptide is in the context of the protein but not when the peptide is isolated (Figure S6). The redesigned sequences also exhibit a preference for charged residues (mostly Asp, Glu, and Lys) in at least two positions (Figure S5). These charged residues, we believe, are what attributes the redesigned peptides their specificity to CRIP1. In particular, the designed sequence A1M (Figure S5), which is a member of the largest cluster in the CRIP1 and 1-ns peptide complexes, exhibits a preference for either Lys or Glu in the 2^nd^ position, Asp in the 5^th^, Lys in the 7^th^, and Gly in the rest.

A closer inspection of the specific energy contributions to the ΔΔ*G* of A1M (Table S2), we found that the largest contributions to ΔΔ*G* arises from more favorable van der Waals interaction between the peptide and CRIP1, which we believe is reflected in the preference for Gly in some sites of the binding peptide. The CRIP1-peptide complex also exhibits more favorable solvation energy after the redesign. This observation is also reflected structurally in Figure 3. In particular, the peptide side chains in A1 (such as 4D, 5N, 6H, and 8S) that point toward the CRIP1 surface are replaced by Gly, while those pointing to solution (3K and 7R) retain their polar nature.

### Binding Affinity

**Figure 4:**
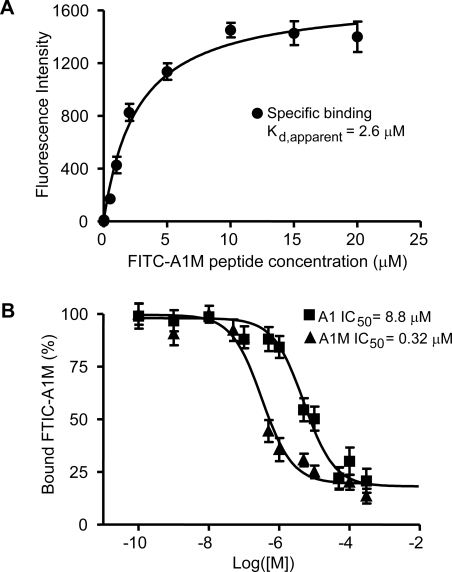
Binding affinity of the ligand peptides to CRIP1 from saturation binding (apparent *K*_d_) and from competitive binding (IC_50_). (A) The apparent *K*_d_ for binding of FITC-A1M to CRIP1 protein was determined by a saturation binding experiment using 1 mM of unlabeled A1M peptide to assess non-specific binding, K_d apparent_  =  2.6 uM. Error bars represent the S.E. of the corrected mean. (B) To compare the binding affinity of A1M and A1 to CRIP1 we performed a competitive binding assay. The concentration of the labeled ligand (FITC-A1M) was held constant and increasing concentrations of either unlabeled A1M or unlabeled A1 peptides were used to compete the binding. From these binding curves regression analysis was used to calculate the IC_50_ for each of the competitors. Both peptides competed off FITC-A1M suggesting that there is only a single binding site for this peptide on the CRIP1. A1M was approximately 27.5 times more effective than A1 at competing for FITC-A1M binding to CRIP1. Error bars represent S.E. of the corrected mean.

We computationally redesigned A1 resulting in a peptide with a new sequence (denoted as A1M) predicted to bind to CRIP1 with higher affinity. To test this prediction, A1, B5 and A1M peptides were all synthesized and labeled with FITC for binding studies. Since the peptides encoded by the C7C phage library are at the N-terminus of the minor phage coat protein pIII followed by a short phage encoded spacer Gly-Gly-Gly-Ser, we included this 4-mer in the synthesized peptide. An additional C-terminal Lys was also included in order to enable fluorescent labeling of the peptide. Thus, the different selected mimotopes were produced as synthetic peptides with Gly-Gly-Gly-Ser-Lys and then labeled by adding a fluorescent molecule to the C-terminal lysine. We synthesized the cyclic form of the peptides, A1, B5 and A1M and determined their ability to bind CRIP1 using saturation binding experiments. The value for the apparent equilibrium dissociation constant (*K*_d apparent_) of the FITC-A1M peptide determined by saturation binding was 2.6 µM, Figure 4A. This was substantially lower than that obtained for either the parent A1 peptide (*K*_d apparent_  =  34.4 µM) or the estimate for the *K*_d apparent_ of the B5 peptide (*K*_d apparent_  =  62.5 µM) derived using similar assays, data not shown. To directly compare the affinity of the A1 and A1M peptides for CRIP1 protein, we performed a competitive binding assay and determined the IC_50_ for each of the peptides using FITC-A1M as the ligand. These studies demonstrated that the binding affinity of the A1M peptide to CRIP1 was approximately 27.5 times better than that of the original A1 peptide, Figure 4B (A1 peptide IC_50_  =  8.8 µM, A1M peptide IC_50_  =  0.32 µM). Since each peptide was effective at displacing FITC-A1M and reached the same minimal binding these data also suggest that ligand binding to CRIP1 occurs at a single site. Further analysis of the binding data with multiple binding site models clearly showed that the best fit of the data was obtained with a one binding site model. Both experimental results are further supported by the predicted binding sites for each peptide to CRIP1 as depicted in Figure 3. Interestingly, when the *K*_i_ for the A1M peptide is calculated (*K*_i_  =  0.067 µM), it is not the same as the apparent *K*_d_ for FITC-A1M (*K*_d_  =  2.6 µM) determined by saturation binding experiments. Based on this observation, the FITC label likely reduces the affinity of the peptide for CRIP1, which is not uncommon with labeled peptides. However, this observation does not alter the interpretations of the data comparing the affinity of the unlabeled peptides A1 and A1M.

From the apparent equilibrium dissociation constants, we calculate the experimental free energy change to be ΔΔ*G*  =  *RT* ln *K*_d,A1M_ − *RT* ln *K*_d,A1_  =  −1.6 kcal mol^−1^, which is smaller than the estimated computational free energy change ΔΔ*G*  =  −83 kcal mol^−1^ (Table 2). This difference between the experimental and computational free energy changes is primarily contributed by the van der Waals repulsion term (Table S2), suggesting initial clashes in the docking of the A1 peptide to the CRIP1 structure. However, since the docking protocol (ZDOCK) is consistently implemented, we still expect strong correlation between the computational and experimental free energy changes, that is, those redesigned peptides with lower computational ΔΔ*G* is also expected to have low experimental ΔΔ*G*, although the absolute values may not be directly comparable. In a separate study benchmarking the Medusa force field [28],[29], experimental and computational ΔΔ*G* values exhibited a correlation of 0.75 (*P* = 10^−108^).

## Discussion

CRIP1 is an extremely compelling marker to exploit for enhanced detection of breast and other cancers. However, its cytosolic expression makes it hard to measure by conventional means, e.g., antibodies. The cell membrane increases the pharmacological barriers that must be overcome to bind and consequently image the expression of this protein in cancer cells. Thus, we developed methodologies to generate high affinity peptides to purified cytosolic proteins with the ultimate aim of designing these peptides to cross membranes and serve as imaging ligands. To rapidly identify peptides that will bind to CRIP1, we utilized phage display technology and purified CRIP1 protein. This technology identifies relatively low affinity (10–100 µM) ligands to target proteins. To increase the affinity of the peptides identified using phage display, we developed a protocol for rational peptide redesign that utilizes computational techniques. This protocol successfully increased peptide affinity by approximately 10–28-fold.

Computational design methods have been employed to modulate protein-protein interactions. Major challenges in protein design include (1) identification of ligand-peptide binding site and (2) optimization of affinity of the peptides that bind to that particular protein [30]. In practice, sequence and conformational space need to be adequately sampled [30]. There is also the need for accurate energy functions that identifies protein sequences corresponding to the global free energy minimum of a given protein conformation [30]. Several studies have been reported to identify protein interaction specificity [31]–[35]. For example, Shifman and Mayo computationally redesigned the promiscuous binding site of calmodulin to increase its specificity to one of its ligand peptides [36]. The authors performed iterative optimization of the rotamers. In another study, Reina et al. computationally engineered a small protein-protein interaction motif of the PDZ domain to bind novel target sequences [37]. The study demonstrated that by combining different backbone templates with computer-aided protein design, PDZ domains could be engineered to specifically recognize a large number of proteins [37]. Another example of successful redesign was the engineering of coiled-coil interfaces that direct the formation of either homodimers or heterodimers [38]. The design protocol involved both positive design, stabilization of desired interaction, and negative design, the destabilization of undesired interactions [38].

The problem of redesigning ligand peptides initially identified from phage display is challenging because the structure of the peptides are not known and the peptides do not have a known binding site in CRIP1. While there have been successes in the redesign of protein-protein interfaces and location of binding site through computational docking, there is yet no study where the system being designed face these two major challenges simultaneously. We computationally modeled the cyclic peptide and performed molecular dynamics to find the equilibrium conformation of the peptide. To diversify the backbone conformation of the peptide included in the redesign, we selected 3 peptides from the equilibrium molecular dynamics and docked them to 48 CRIP1 conformations from NMR. Interestingly, this procedure of diversifying protein and ligand peptide conformation is sufficient to identify putative binding sites on the protein. We believe that the cyclic structure of the peptide was an important factor to the success of the procedure, because the error from enthalpy-entropy compensation is reduced when docking a cyclic peptide compared to docking a linear peptide.

Another important factor that contributed to the success of the peptide design is the conformational sampling introduced in the design steps to maximize the coverage of sequence-structure space available to the CRIP1-peptide complex. First, we performed multiple docking simulations that allowed us to identify various poses for binding. Second, we allowed backbone of the peptide to be flexible during sequence design procedure, thereby significantly diversifying the designed sequences [28],[29]. Hence, the combination of the restricted conformational space available to a peptide due to circularization and our flexible-backbone sampling technique [28],[29] allowed us to sufficiently sample the conformational space of the peptide during design, thereby contributing to a successful peptide binder to CRIP1. Our approach can be further extended to other systems of interest.

In this study, we combine empirical and computational approaches to develop a novel paradigm to improve ligand affinity when limited structural information is available. CRIP1, a potentially powerful biomarker for several cancers, was purified and used in an empirical phage display assay to identify short amino acid peptides with modest affinity for the protein. The resulting peptides were then structurally modeled, based on the structures of other known but unrelated peptides of similar size. Using the limited NMR structure available for CRIP1 the modeled peptides were then computationally docked to CRIP1 resulting in identification of several potential structural motifs responsible for the binding interaction. The modeled interactions were then optimized and peptides were redesigned based on these data.

Interestingly, even after 4 rounds of phage display isolation, no consensus sequence for CRIP1 binding peptides emerged. These data might possibly suggest that a strong binding “natural” peptide did not exist on the CRIP1 protein. Remarkably, however, computational manipulation of the amino acids contained within the peptide, based on energy minimization, significantly increased the affinity of the peptide. This suggests that: (1) conditions for phage-CRIP1 binding were not optimal for peptide identification; (2) the phage library did not contain all possible combinations of amino acids; and/or (3) the library was not exhaustively screened. In any of these cases, however, the use of computational redesign combined with empirically derived initial binding data significantly improved the quality of final peptide ligands. As our database of redesigned peptides and resulting *K*_d_'s accumulates, the approaches described here potentially can be generalized and could be implemented for peptide ligand generation routinely. The resulting peptide from these studies, A1M, will be further developed as an imaging probe.

## Methods

### Construction of Vectors pHat10-CRIP1 and Transformation into *E. coli*

The coding region of the CRIP1 cDNA was removed from CRIP1 in pcDNA3.1+ using BamH1 and Xba1 restriction enzymes and subsequently subcloned into the BamHI and EcoRI sites of the vector pHAT10 (BD Clontech) which contains an N-terminal histidine affinity tag. The construct was confirmed by sequencing.

### CRIP1 Protein Expression and Purification

Bacterial cells expressing the pHAT10-CRIP1 were cultured in LB media containing 50 µg/ml ampicillin until reaching OD of 0.6 at which time they were induced to express the protein by adding IPTG to a final concentration of 0.5 mM IPTG. The bacteria were then harvested and resuspended in Equilibration/Wash Buffer (50 mM sodium phosphate pH 7.0, 300 mM NaCl) containing 0.75 mg/ml lysozyme and 0.0174 mg/ml PMSF and sonicated with three 10 s pulses (medium power, Sonic Dismembrator Model 100, Fisher Scientific), with a pause for 30 s on ice between sonication cycles. Following sonication, the lysates were cleared by centrifugation, and incubated with TALON CellThru Resin (BD Biosciences, Palo Alto, CA) in Extraction/Wash Buffer. The tagged protein was eluted from the washed column with 0.15 M imidazole in Extraction/Wash Buffer. The purity of CRIP1 in fractions was confirmed by SDS-PAGE [39]. The concentration of CRIP1 in fractions was determined by Bradford Assay using IgG as a standard [40].

### Phage Display

CRIP1 was digested with enterokinase (Roche Diagnostics, Inc.) to remove the His tag and then was used as bait for 4 rounds of panning with the Ph.D.-C7C Phage Display Peptide Library (New England Biolabs). The nucleotide sequence of the gene III insert was determined by sequencing the phage, and the amino acid sequence of the insert was deduced from the nucleotide sequence, shown in Table 1.

### Molecular Modeling and Redesign

Computational optimization of the peptide binding affinities consists of three major steps: (1) structural modeling of cyclic peptides initially identified from phage display experiments, (2) finding putative binding sites of the peptides on CRIP1, and (3) searching for sequences that optimize the stability of the peptide-CRIP1 complex.

#### Peptide model

We first constructed a linear peptide model of A1. To circularize the linear peptide, we assigned a disulfide bond between the sulfur atoms of the terminal cysteines and performed rapid descent energy minimization. Peptide modeling was performed in InsightII (Accelrys, San Diego, CA), a molecular modeling suite.

To further relax the structure of the cyclic peptide, we performed all-atom 10 ns equilibrium molecular dynamics simulation of A1 in GROMACS [41],[42] (see Figure 1C for cyclic peptide structure after 10 ns simulation.) The peptide was solvated in a rectangular box filled with SPC water molecules [43]. A chloride ion was added to the system such that the net charge of the system is zero. OPLSAA force field was used to define interactions between protein atoms [44]. We employed the Particle Mesh Ewald (PME) method to calculate the electrostatics interactions in the system [45],[46]. The system was coupled to an external thermal bath at 300 K with a coupling constant of *τ*_T_  =  0.1 ps [47]. The system pressure was also maintained at 1.0 bar by an isotropic pressure coupling with time constant τ_P_  =  0.5 ps [47]. In both the peptide redesign and identification of binding sites, we selected the peptide conformations from the equilibrium simulation corresponding to 1 ns, 9 ns, and 10 ns, which are labeled as 1-ns, 9-ns, and 10-ns, respectively.

#### Putative binding sites on CRIP1

To increase the binding affinity of the initially identified peptide A1, we needed structures of peptides docked to CRIP1. To arrive at the CRIP1-peptide complexes, we docked the three peptides to the 48 CRIP1 conformations derived from NMR (Figure 2). CRIP1 structure contains a long unstructured N-terminal loop, which include residues G61 to K76 (Figure 1A). Peptides that docked exclusively to this loop were excluded in the redesign. We used ZDOCK to find candidate peptide binding sites on CRIP1 [48],[49]. ZDOCK performs a fast Fourier transform search of all possible binding modes for proteins based on shape complementarity, desolvation energy, and electrostatics [48],[49].

For each candidate peptide, we identified the dominant binding modes by clustering them according to their position on the CRIP1 surface (Figure 2). We first defined the position of the peptide by the center of mass of its C_α_ atoms. Then, using a hierarchical clustering algorithm, we were able to group the centroids. To find the optimal number of clusters, we first varied the cutoff (maximum distance between two subnodes that belong to the same cluster) (Figure 2). In clustering, there are two competing parameters, the number of clusters and the similarity between elements within a cluster. In the maximum number of clusters, each element is itself a cluster, and the single element is perfectly similar to itself. However, this limit does not reveal the underlying structure of the data points. In the opposite limit where we have only one cluster, all objects belong to the same cluster, which is still not informative. But as shown in Figure 2, the optimal balance between number of clusters and similarity is attained when the cutoff is 6.9 Å for 1-ns peptides. For the two other peptides, the cutoffs were 6.4 and 7.4 Å, respectively.

We show in Figure 2 the positions of the peptides that were docked to CRIP1. Docking sites that belong to the same cluster are colored similarly. The redesigned peptide A1M, belongs to the largest cluster.

#### Peptide redesign

All the CRIP1-peptide complexes derived from the docking were subjected to redesign. We optimized the binding of the peptide by computationally mutating each peptide residue and searching for the peptide sequence with low ΔΔ*G* = Δ*G*_MUT_ −Δ*G*_A1_, where Δ*G*_MUT_ and Δ*G*_A1_ are the free energies of the redesigned peptide and original peptide A1, respectively. The detailed methodology of the computational ΔΔG estimation is described in an earlier study [28],[29],[50] (see also the freely accessible server for the ΔΔ*G* estimation ERIS, http://dokhlab.unc.edu/tools/eris/index.html). ERIS uses a united atom model, which includes all heavy atoms and polar hydrogen atoms, to represent proteins. ERIS likewise employs a physical force field (called Medusa [51]) coupled with fast side-chain packing and backbone relaxation algorithms. The calculated free energy is a weighted sum of van der Waals interaction, solvation energy, hydrogen bonding, and backbone-dependent statistical energy for any given amino acid and rotamer state. The ERIS ΔΔ*G* estimation protocol has been benchmarked in earlier study [28],[29] by comparing calculated free energy changes with experimental values.

We ranked according to ΔΔ*G* values the peptide sequences that were redesigned from the same backbone conformation and were docked on the same cluster of binding sites. In Table 2, we show some representative sequences from the peptide redesign. In these calculations, the peptide A1M (CLDGGGKGC) exhibited both a high binding mode rank (most other A1 peptides docked to the same site) and a low ΔΔ*G* energy, which we selected as the candidate for experimental verification.

### Peptide Synthesis

#### Peptide on resin

Peptide was synthesized on a Peptide Synthesizer 433A (Applied Biosystems) using Fmoc chemistry protocols with HBTU activation (please see schema in Figure S4). The starting resin was Fmoc-Rink-Amide resin (Elim Biopharmaceuticals) or Fmoc-Knorr Amide Resin (Case Western Reserve University). All amino acids used standard side chain protecting groups, except for the C-terminal lysine residue, which contained a (4,4-dimethyl-2,6-dioxocyclohex-1-ylidene)ethyl (Dde) functionality protecting the ε-amino group to allow orthogonal synthesis by selective deprotection of the Dde while the peptide was attached to the resin. The N-terminal cysteine residue was protected by a Boc group. To provide a linker and a conjugation site, a Lys and the sequence Gly-Gly-Gly-Ser derived from the phage sequences immediately downstream of the C7C insert, were added to the synthesized peptides. After completion of the synthesis, the peptide resin was washed with DIPEA and DCM and kept dry for the next reaction.

#### FITC-peptide on resin

The Dde protecting group was removed by suspending the resin in 2% hydrazine monohydrate in DMF (25∼100 ml/g, 3∼10 times×3∼60 min). After thorough washing with DMF and methanol, 2 eq of 5-carboxyfluorescein was added with 2 eq of TBTU, 2 eq of HOBt, and 8 eq of diisopropylethylamine in NMP. The coupling of 5-carboxyfluorescein was allowed to proceed for 24 h at room temperature. The FITC-peptide on resin was then washed with NMP, methanol, and kept dry for the next reaction [52].

#### FITC-peptide

FITC-peptide was cleaved from the resin support using 2.5% EDT, 1% TIS, 94.5% TFA and 2.0% water for 2 h at room temperature and precipitated in ether. The FITC-peptide was purified by reverse phase HPLC (Shimazu LC-20AT, SPD-10 UV detector) on a Luna 5 µ C18(2) column (250 mm×10 mm, Phenomonex Corp.) using a linear gradient system of 0.1% TFA aqueous solution with an initial concentration of acetonitrile 5%. The calculated mass was confirmed by mass spectrometry (PE Biosystem, ProTOF ). FITC-A1 peptide with sequence NH_2_-C-L-K-D-N-H-R-S-C-G-G-G-S-K-(FITC)-CONH_2_: *m/z*: 1818.7; calculated mass: C_77_H_107_N_23_O_25_S_2_, 1817.7. FITC-B5 peptide with sequence C-Y-D-P-I-W-R-T-C-G-G-G-S-K-(FITC)-CONH_2_: *m/z*: 1898.6; calculated mass: C_87_H_110_N_20_O_25_S_2_, 1897.6. FITC-A1M peptide with sequence NH_2_-C-L-D-G-G-G-K-G-C-G-G-G-S-K-(FITC)-CONH_2_: *m/z*: 1552.6; calculated mass: C_66_H_89_N_17_O_23_S_2_, 1551.0.

#### Cyclization of FITC-peptide [53]

FITC-peptide was resuspended at 0.5-1 mg/ml and oxidized in 10∼20% DMSO aqueous solution adjusted to pH 7 by (NH_4_)_2_CO_3_. At the completion of the reaction, usually 4∼10 hours, the solution was purified by reverse phase HPLC (Shimazu LC-20AT, SPD-10 UV detector) on a Luna 5 µ C18(2) column (250 mm×10 mm, Phenomonex Corp.) using a gradient system of 0.1% aqueous TFA with an initial concentration of acetonitrile 5%. The calculated mass was confirmed by mass spectrometry (PE Biosystem, ProTOF). FITC-cyclic-A1 peptide with sequence NH_2_-C-L-K-D-N-H-R-S-C-G-G-G-S-K-(FITC)-CONH_2_: *m/z*: 1816.8; calculated mass: C_77_H_105_N_23_O_25_S_2_, 1815.7. FITC-cyclic-B5 peptide with sequence C-Y-D-P-I-W-R-T-C-G-G-G-S-K-(FITC)-CONH_2_: *m/z*: 1896.6; calculated mass: C_87_H_108_N_20_O_25_S_2_, 1895.6. FITC-cyclic-A1M peptide with sequence NH_2_-C-L-D-G-G-G-K-G-C-G-G-G-S-K-(FITC)-CONH_2_: *m/z*: 1550.6; calculated mass: C_66_H_87_N_17_O_23_S_2_, 1549.0. The synthesis was shown in Scheme 1. Circularization resulted in a loss of 2 protons as measured by Mass Spec analysis.

### Measurement of Binding Affinity

The binding affinity of the peptides for CRIP1 protein was determined by saturation binding experiments [54],[55]. Ninety-six well plates were coated with 150 µl of PBS buffer containing 100 µg/ml of CRIP1 and incubated overnight at 4°C. The wells were then washed three times with 50 mM Tris, 150 mM NaCl, pH 7.5 (TBS) containing 0.1% Tween-20 (TBST), and then each well filled completely with blocking buffer (TBS containing 0.5% BSA), incubated at least 1 hour at 4°C, and then rapidly washed 3 times with TBST. Following washing 100 µl of binding buffer containing different concentrations of FITC-labeled peptides (ranging from 50 nM to 100 µM) were added to the CRIP1 containing wells and incubated for 1 hour at 37°C with rocking. After incubation, the plates were washed three times with binding buffer. The fluorescence intensity in each well was determined on Infinite M200 Tecan Instrument (Tecan, NC) (Excitation wavelength: 494 nm, Emission wavelength: 530 nm). The apparent equilibrium dissociation constant, *K*_d,apparent_, was calculated by non-linear regression using GraphPad Prism (GraphPad Prism 4.0 Software, San Diego, CA). Each data point is the average of three determinations. Nonspecific binding was defined in the presence of 1 mM unlabeled peptide. All binding experiments (saturation binding and competitive binding experiments) were conducted under equilibrium binding conditions and under conditions where total ligand added was essentially equivalent to the amount of free ligand after the binding reaction occurred.

### Competitive Binding Assay

The binding affinity of the A1 and A1M peptides for CRIP1 protein was directly compared by a competitive binding experiment [55],[56]. Labeled A1M peptide (FITC-A1M) was competed with increasing concentrations of either unlabeled A1M peptides or A1 peptide and the IC_50_ for each peptide calculated.

Ninety-six well plates were coated with 150 µl of PBS buffer containing 100 µg/ml of CRIP1 and incubated overnight at 4°C. The wells were then washed 3 times with 50 mM Tris, 150 mM NaCl, pH 7.5 (TBS) containing 0.1% Tween-20 (TBST), and then each well filled completely with blocking buffer (TBS containing 0.5% BSA), incubated at least 1 hour at 4°C, and then rapidly washed 3 times with TBST. Following washing 150 µl of binding buffer containing FITC-A1M peptides of 10 µM and appropriate dilutions of unlabeled A1 and A1M peptides (ranging from 0 to 300 µM) were added to the CRIP1 containing wells and incubated for 1 hour at 37°C with rocking. After incubation, the plates were washed three times with binding buffer. The fluorescence intensity in each well was determined on Infinite M200 Tecan Instrument (Tecan, NC) (Excitation wavelength: 494 nm, Emission wavelength: 530 nm). Ki was calculated by non-linear regression with one binding site using GraphPad Prism (GraphPad Prism 4.0 Software, San Diego, CA). Each data point is the average of three determinations, shown in Figure 4B. Data was analyzed using several different binding models and was found to only fit a one binding site model. When no competitor was added the data point was graphed as 0.1 nM to satisfy software requirements. This has no effect on calculations of the IC_50_'s.

## Supporting Information

Figure S1 The cDNA and amino acid sequences of CRIP1. After cloning, the insert was confirmed by sequencing and the deduced amino acid sequences for the human CRIP1 shown in Figure 1S. Excluding the vector sequence and the poly A region, the cDNA insert is 243 base pairs in length. The start site for transcription is at nucleotide position 73 (not shown in figure) with the start of translation at nucleotide position 162. This open reading frame expresses the amino acids encoding the His-tag (nt: 186–242) and encoding an enterokinase clevage site (nt: 246–260). The sequences encoding the human CRIP1 protein begin at nucleotide 273 and continue through nucleotide 503. Translation of these sequences results in a polypeptide 114 amino acids in length, the majority of which, 77 amino acids, make up CRIP1 protein. The start (ATG) and stop (TAA) codons are underlined. The sequence of nonadjacent 6 histidines on HAT epitope is in bold. The poly A tail at the end is not shown.
(0.80 MB TIF)

Figure S2 CRIP1 purity. Comassie Blue stained-SDS-PAGE analysis of CRIP1 lysate and elutions after purification. Lane 1: standard molecular marker; Lane 2: lysate before incubation with Resin; Lane 3: lysate after incubation with Resin; Lane 4∼5: fractions through Clontech TALON CellThru column.
(1.30 MB TIF)

Figure S3 Relative estimates of peptide affinity for CRIP1. Phage binding against immobilized CRIP-1.
(0.38 MB TIF)

Figure S4 Synthesis of FITC-peptides. Please see Methods for detailed description.
(0.92 MB TIF)

Figure S5 Sequences of redesigned peptides. Sequence motifs of the redesigned peptides for the starting peptide structure models 1-ns, 9-ns, and 10-ns. The rank pertains to the order putative the binding site on CRIP1 defined from clustering.
(0.68 MB TIF)

Figure S6 Residue preference without CRIP context. To verify that the observed preference for Gly in some sites in the peptide is not due to a bias in the force field, we employed the protocol to find the optimal peptide sequence when the peptide is not bound to CRIP1. We used 50 independent redesign runs. The preferred sequences are expectedly highly polar which maximize the peptide solvation energy.
(0.09 MB TIF)

Table S1 Analysis of CRIP1.
(0.04 MB DOC)

Table S2 Contribution of individual energy terms to the ΔΔG of the redesigned peptide A1M CLDGGGKGC.
(0.04 MB DOC)
