# Supplementary figures and images for: Identification and Rational Redesign of Peptide Ligands to CRIP1, A Novel Biomarker for Cancers
Source: PLoS Comput Biol. 2008 Aug 1;4(8):e1000138. doi: 10.1371/journal.pcbi.1000138 (PMC2453235; doi:10.1371/journal.pcbi.1000138)

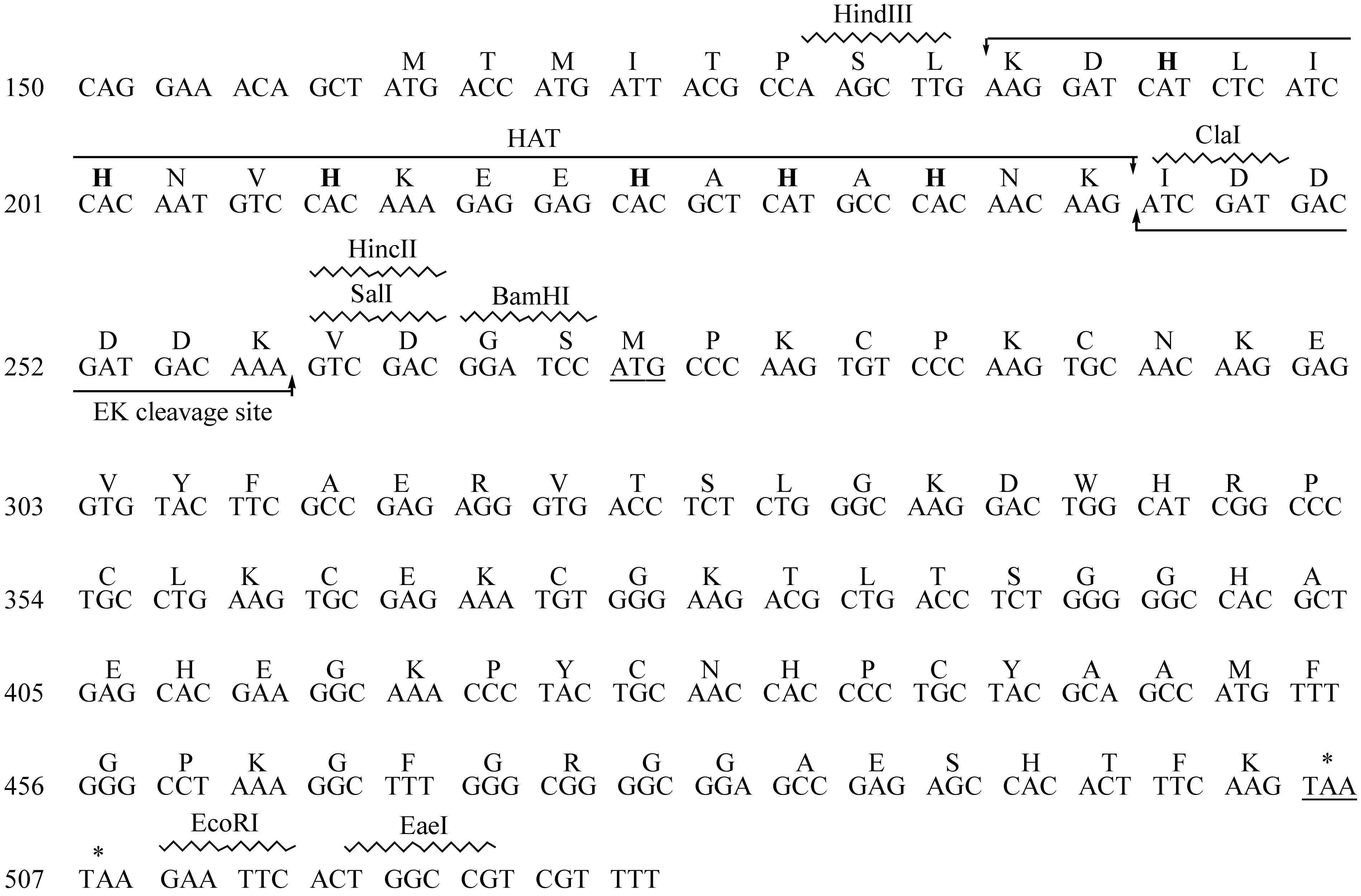

Supplement: Figure S1 — The cDNA and amino acid sequences of CRIP1. After cloning, the insert was confirmed by sequencing and the deduced amino acid sequences for the human CRIP1 shown in Figure 1S. Excluding the vector sequence and the poly A region, the cDNA insert is 243 base pairs in length. The start site for transcription is at nucleotide position 73 (not shown in figure) with the start of translation at nucleotide position 162. This open reading frame expresses the amino acids encoding the His-tag (nt: 186–242) and encoding an enterokinase clevage site (nt: 246–260). The sequences encoding the human CRIP1 protein begin at nucleotide 273 and continue through nucleotide 503. Translation of these sequences results in a polypeptide 114 amino acids in length, the majority of which, 77 amino acids, make up CRIP1 protein. The start (ATG) and stop (TAA) codons are underlined. The sequence of nonadjacent 6 histidines on HAT epitope is in bold. The poly A tail at the end is not shown. (0.80 MB TIF) [file pcbi.1000138.s001.tif]

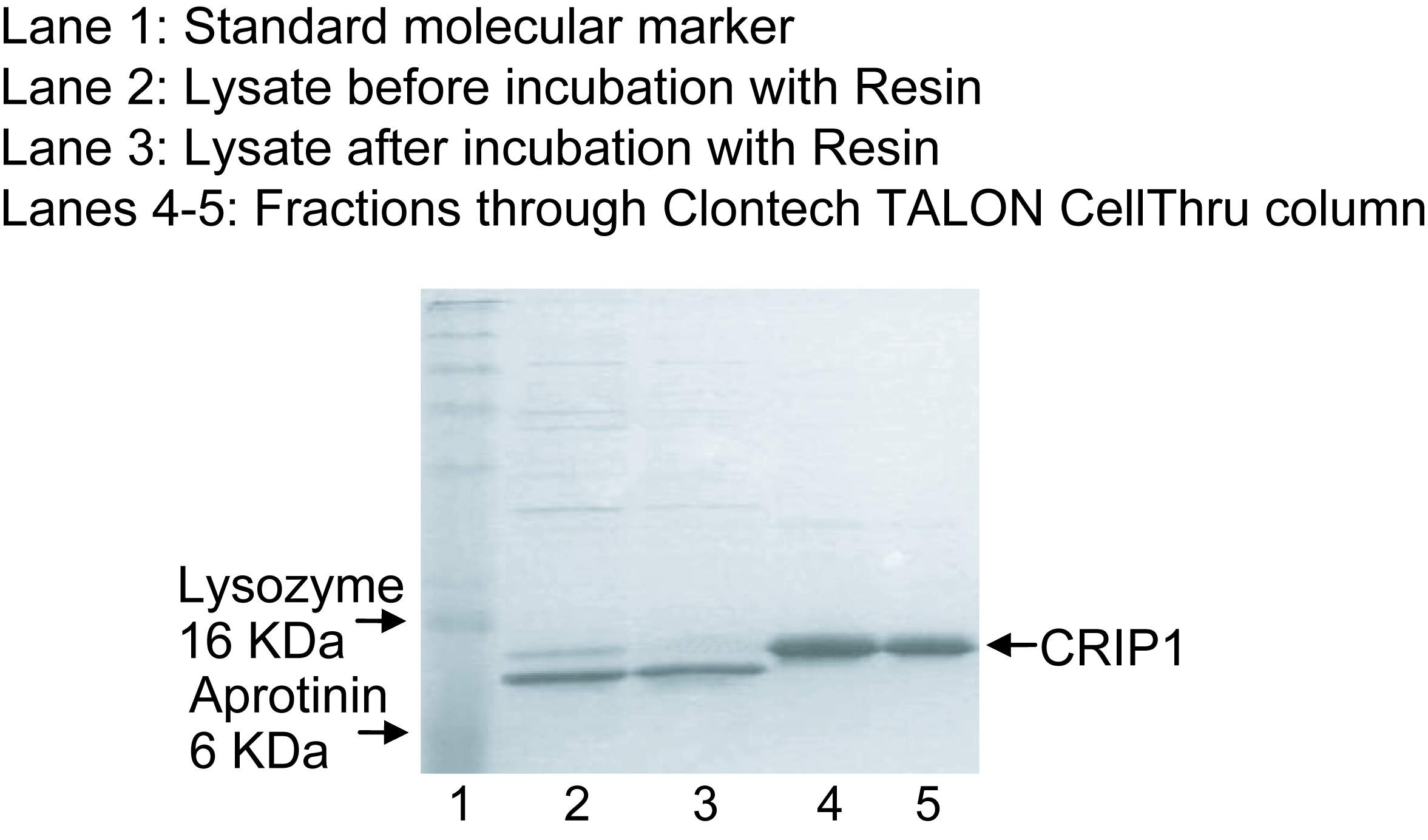

Supplement: Figure S2 — CRIP1 purity. Comassie Blue stained-SDS-PAGE analysis of CRIP1 lysate and elutions after purification. Lane 1: standard molecular marker; Lane 2: lysate before incubation with Resin; Lane 3: lysate after incubation with Resin; Lane 4∼5: fractions through Clontech TALON CellThru column. (1.30 MB TIF) [file pcbi.1000138.s002.tif]

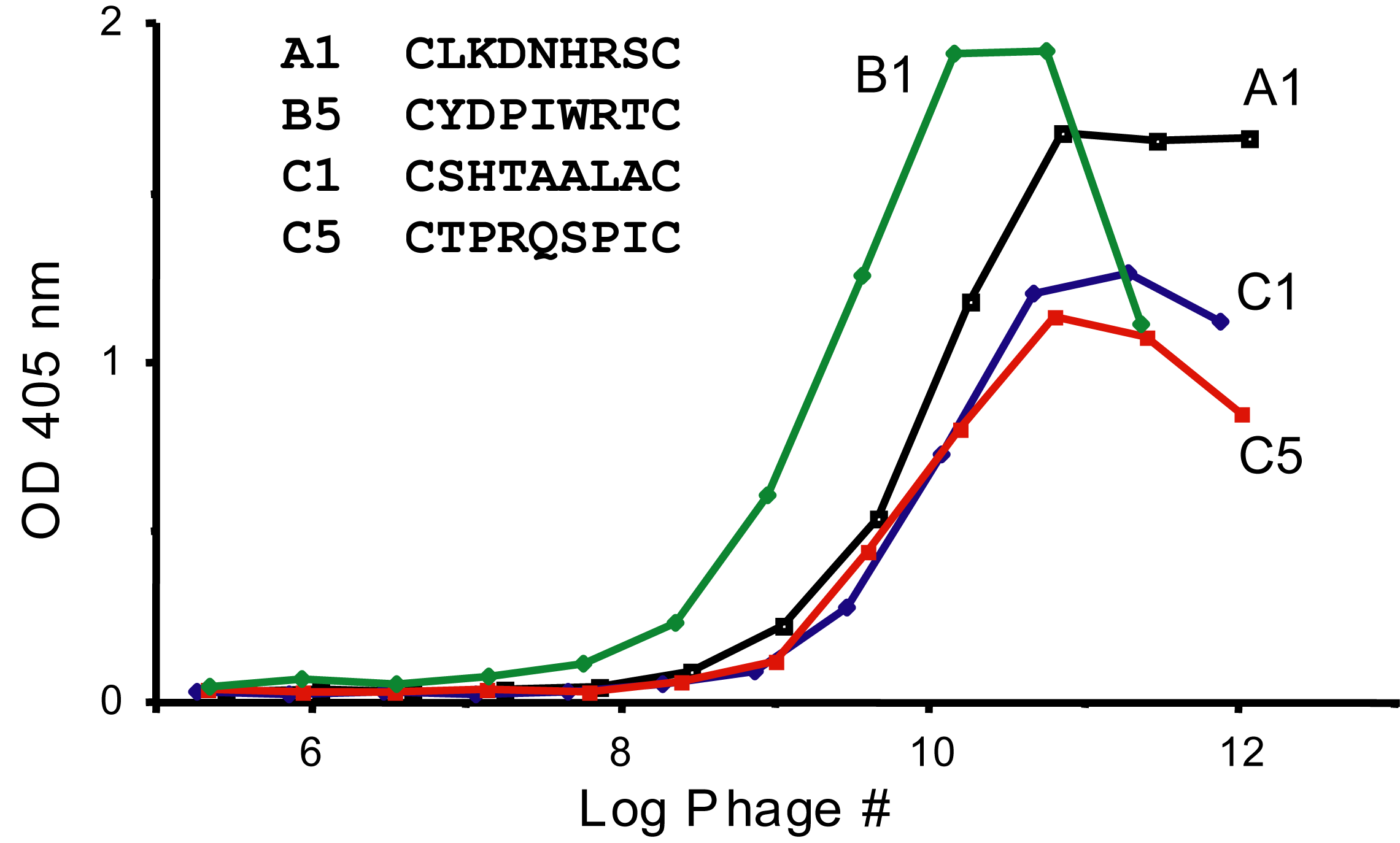

Supplement: Figure S3 — Relative estimates of peptide affinity for CRIP1. Phage binding against immobilized CRIP-1. (0.38 MB TIF) [file pcbi.1000138.s003.tif]

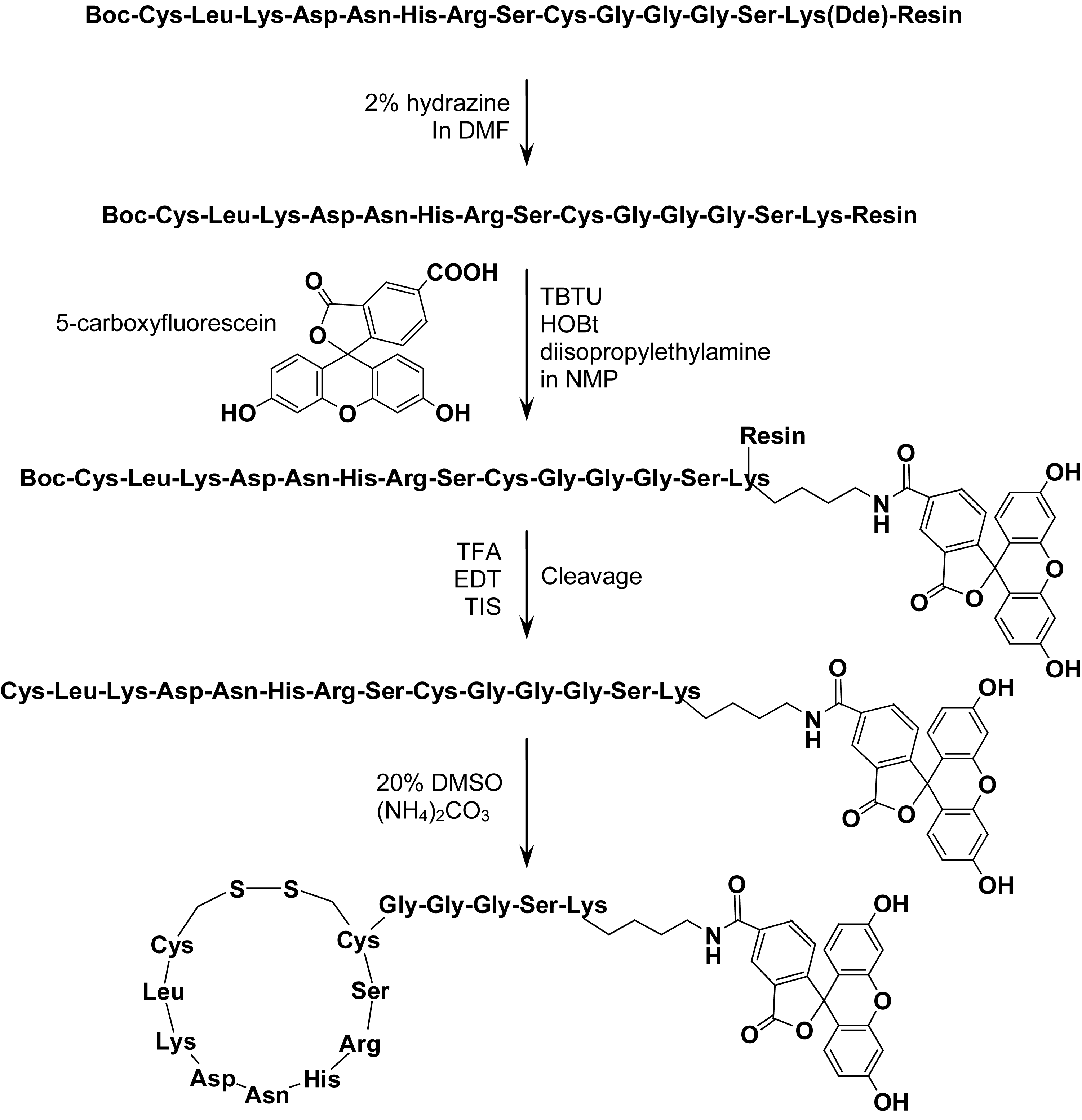

Supplement: Figure S4 — Synthesis of FITC-peptides. Please see Methods for detailed description. (0.92 MB TIF) [file pcbi.1000138.s004.tif]

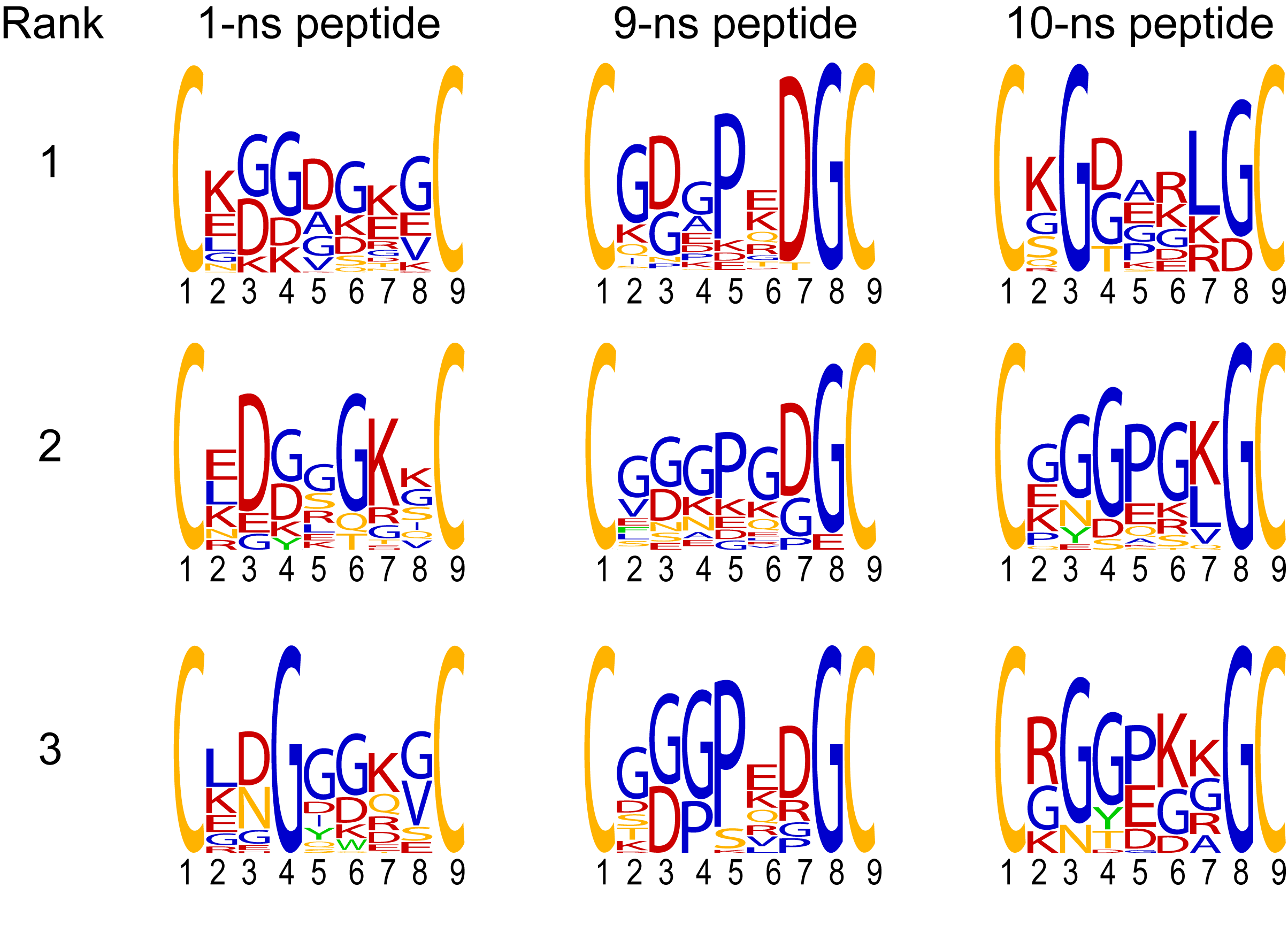

Supplement: Figure S5 — Sequences of redesigned peptides. Sequence motifs of the redesigned peptides for the starting peptide structure models 1-ns, 9-ns, and 10-ns. The rank pertains to the order putative the binding site on CRIP1 defined from clustering. (0.68 MB TIF) [file pcbi.1000138.s005.tif]

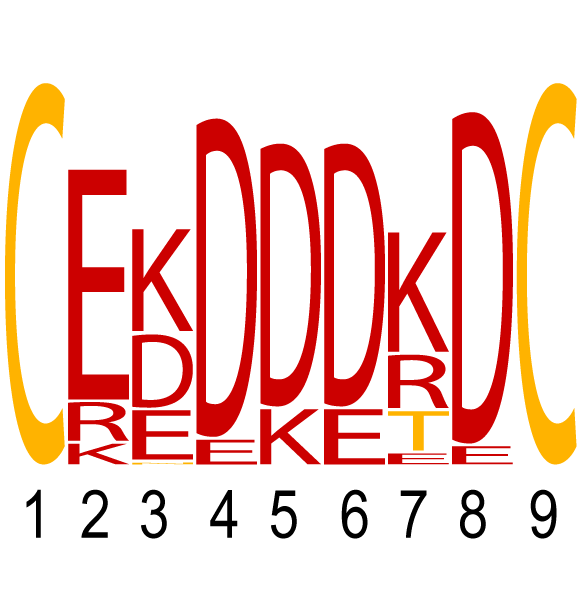

Supplement: Figure S6 — Residue preference without CRIP context. To verify that the observed preference for Gly in some sites in the peptide is not due to a bias in the force field, we employed the protocol to find the optimal peptide sequence when the peptide is not bound to CRIP1. We used 50 independent redesign runs. The preferred sequences are expectedly highly polar which maximize the peptide solvation energy. (0.09 MB TIF) [file pcbi.1000138.s006.tif]
