# Supplementary material for: Identification and Rational Redesign of Peptide Ligands to CRIP1, A Novel Biomarker for Cancers
Source: PLoS Comput Biol. 2008 Aug 1;4(8):e1000138. doi: 10.1371/journal.pcbi.1000138 (PMC2453235; doi:10.1371/journal.pcbi.1000138)
Supplement: Table S1 — Analysis of CRIP1. (0.04 MB DOC) [file pcbi.1000138.s007.doc]

| **Analysis** | **Entire Protein** |
| --- | --- |
| Length | 114 aa |
| Molecular Weight | 12743.72 |
| 1 microgram | 78.470pMoles |
| Molar Extinction coefficient | 10370 |
| 1 A[280] corr. To | 1.23 mg/ml |
| A[280] of 1 mg/ml | 0.81 AU |
| Isoelectric point | 8.20 |
| Charge at pH 7 | 2.66 |
