# Supplementary material for: Identification and Rational Redesign of Peptide Ligands to CRIP1, A Novel Biomarker for Cancers
Source: PLoS Comput Biol. 2008 Aug 1;4(8):e1000138. doi: 10.1371/journal.pcbi.1000138 (PMC2453235; doi:10.1371/journal.pcbi.1000138)
Supplement: Table S2 — Contribution of individual energy terms to the ΔΔG of the redesigned peptide A1M CLDGGGKGC. (0.04 MB DOC) [file pcbi.1000138.s008.doc]

| **Energy Term^a^** | **Symbol** | **Value (kcal/mol)** |
| --- | --- | --- |
| van der Waals attraction | evdw_a | 17.3 |
| van der Waals repulsion | evdw_r | -99.1 |
| Solvation | esolv | -15.6 |
| Hydrogen bond interactions between sidechain and backbone | ehb_sb | 1.1 |
| Hydrogen bond interactions between sideschains | ehb_ss | 0.0 |
| Internal energy for a rotamer state | efy_chi | 1.0 |
| Correction for dependence rotamer energy on backbone conformation and amino acid type | efy_aa | 2.7 |
| Reference energy^b^ | e_aa_ref | 10.0 |
| **Total ΔΔG** |  | **-83** |

^a^Please see Ding and Dokholyan^51^ for detailed calculation of the force field terms.

^b^Reference energy assumes that the polypeptide is completely unfolded.
